# Genomic imbalances in the placenta are associated with poor fetal growth

**DOI:** 10.1186/s10020-020-00253-4

**Published:** 2021-01-07

**Authors:** Giulia F. Del Gobbo, Yue Yin, Sanaa Choufani, Emma A. Butcher, John Wei, Evica Rajcan-Separovic, Hayley Bos, Peter von Dadelszen, Rosanna Weksberg, Wendy P. Robinson, Ryan K. C. Yuen

**Affiliations:** 1 BC Children’s Hospital Research Institute, 950 W 28th Ave, Vancouver, V5Z 4H4 Canada; 2 Department of Medical Genetics, University of British Columbia, 4500 Oak St, Vancouver, V6H 3N1 Canada; 3 Genetics and Genome Biology Program, The Hospital for Sick Children, 686 Bay St, Toronto, M5G 0A4 Canada; 4 The Centre for Applied Genomics, Genetics and Genome Biology, The Hospital for Sick Children, 686 Bay St, Toronto, M5G 0A4 Canada; 5 Department of Pathology and Laboratory Medicine, University of British Columbia, 2211 Wesbrook Mall, Vancouver, V6T 2B5 Canada; 6 Department of Perinatology, Victoria General Hospital, 1 Hospital Way, Victoria, V8Z 6R5 Canada; 7 Department of Obstetrics & Gynecology, University of British Columbia, Suite 930, 1125 Howe St, Vancouver, BC V6Z 2K8 Canada; 8 Department of Women and Children’s Health, School of Life Course Sciences, King’s College London, London, SE1 7EU UK; 9 Department of Molecular Genetics, Institute of Medical Sciences, University of Toronto, 1 King’s College Circle, Toronto, M5S 1A8 Canada; 10 Department of Molecular Genetics, University of Toronto, 1 King’s College Circle, Toronto, M5S 1A8 Canada; 11 Division of Clinical and Metabolic Genetics, The Hospital for Sick Children, Suite 940, 525 University Avenue, Toronto, ON M5G 1X8 Canada; 12 Department of Paediatrics, University of Toronto, 555 University Avenue, Toronto, ON M5G 1X8 Canada

**Keywords:** Aneuploidy, Confined placental mosaicism, Copy number variant, Fetal growth restriction, Placenta, Pregnancy, Small-for-gestational age, Trisomy

## Abstract

**Background:**

Fetal growth restriction (FGR) is associated with increased risks for complications before, during, and after birth, in addition to risk of disease through to adulthood. Although placental insufficiency, failure to supply the fetus with adequate nutrients, underlies most cases of FGR, its causes are diverse and not fully understood. One of the few diagnosable causes of placental insufficiency in ongoing pregnancies is the presence of large chromosomal imbalances such as trisomy confined to the placenta; however, the impact of smaller copy number variants (CNVs) has not yet been adequately addressed. In this study, we confirm the importance of placental aneuploidy, and assess the potential contribution of CNVs to fetal growth.

**Methods:**

We used molecular-cytogenetic approaches to identify aneuploidy in placentas from 101 infants born small-for-gestational age (SGA), typically used as a surrogate for FGR, and from 173 non-SGA controls from uncomplicated pregnancies. We confirmed aneuploidies and assessed mosaicism by microsatellite genotyping. We then profiled CNVs using high-resolution microarrays in a subset of 53 SGA and 61 control euploid placentas, and compared the load, impact, gene enrichment and clinical relevance of CNVs between groups. Candidate CNVs were confirmed using quantitative PCR.

**Results:**

Aneuploidy was over tenfold more frequent in SGA-associated placentas compared to controls (11.9% vs. 1.1%; *p* = 0.0002, OR = 11.4, 95% CI 2.5–107.4), was confined to the placenta, and typically involved autosomes, whereas only sex chromosome abnormalities were observed in controls. We found no significant difference in CNV load or number of placental-expressed or imprinted genes in CNVs between SGA and controls, however, a rare and likely clinically-relevant germline CNV was identified in 5.7% of SGA cases. These CNVs involved candidate genes *INHBB*, *HSD11B2*, *CTCF*, and *CSMD3*.

**Conclusions:**

We conclude that placental genomic imbalances at the cytogenetic and submicroscopic level may underlie up to ~ 18% of SGA cases in our population. This work contributes to the understanding of the underlying causes of placental insufficiency and FGR, which is important for counselling and prediction of long term outcomes for affected cases.

## Background

Fetal growth restriction (FGR), where the fetus does not grow to its genetic potential, affects 5–12% of pregnancies in developed countries (Kramer 2003). FGR is associated with increased risk for perinatal, neonatal, pediatric and long-term adult health complications (Barker et al. 1993; Bernstein et al. 2000; Leitner et al. 2000; Breeze and Lees 2007; Beckerath et al. 2013). Often small-for-gestational age (SGA, birth weight < 10th percentile) is used as a surrogate for FGR, however, a subset of SGA infants may be small but normally grown for their potential and thus otherwise healthy. In particular, pathologically growth-restricted infants are at increased risk for morbidity and mortality.

Poor growth in utero is most commonly attributed to placental insufficiency, however fetal infection or genetic abnormality, and maternal health or lifestyle factors may also play a role (Roberts and Escudero 2012; Burton and Jauniaux 2018; Sharma et al. 2016). Some of these factors (e.g. maternal smoking, infection, obesity) may also contribute to poor trophoblast development and function, thus the etiologies of FGR and placental insufficiency are complex and intertwined. A major known cause of placental insufficiency in a viable pregnancy is confined placental mosaicism (CPM), where some or most cells in the placenta are aneuploid, while the fetus has a predominantly normal diploid chromosome complement. CPM identified prenatally is associated with increased risk for FGR and other pregnancy complications depending on the levels of abnormal cells and the chromosome(s) involved (Robinson et al. 1997; Grati et al. 2019). Screening placentas postnatally has also confirmed a contribution of CPM to FGR (Artan et al. 1995; Krishnamoorthy et al. 1995; Wilkins-Haug et al. 1995; Stipoljev et al. 2001). We previously identified trisomy CPM in 4/43 FGR pregnancies, but none in 85 controls nor 18 cases associated with preeclampsia (PE) without FGR (Robinson et al. 2010). Despite the evidence that large genomic imbalances in the placenta are associated with FGR, few studies have investigated the role of smaller genetic imbalances (< 5–10 Mb), copy number variants (CNVs). To date, studies investigating CNVs associated with FGR have either not studied placental tissue (Zhu et al. 2016; Borrell et al. 2017) or had small sample sizes and found conflicting results (Kasak et al. 2015; Biron-Shental et al. 2016).

In this study, we aimed to thoroughly evaluate the contribution of placental genomic imbalances to poor fetal growth. To this end, we assessed (i) the incidence of large aneuploidies (> 15 Mb) in 274 placentas from control and SGA pregnancies, and (ii) the load, impact, and clinical relevance of placental CNVs (< 15 Mb) to SGA in a subset of 114 euploid placentas. This is the largest study to date of its kind; it enhances our understanding of the underlying causes of placental dysfunction and poor fetal growth, and further establishes the importance of assessment of CPM in the clinic.

## Methods

### Research ethics approval

Ethics approval for use of human research subjects in this study was obtained from the University of British Columbia/Children’s and Women’s Health Centre of British Columbia Research Ethics board (H17-01545) and from the Hospital for Sick Children (1000038847) and Mount Sinai Hospital (05-0038-E) Research Ethics boards. Informed written consent was obtained from all study participants.

### Sample collection and cohort characteristics

#### Vancouver cohort

Placental samples for the Vancouver cohort were ascertained and processed as described (Robinson et al. 2010) and include cases used in previous studies (Robinson et al. 2010; Yuen et al. 2010; Blair et al. 2013; Wilson et al. 2018; Del Gobbo et al. 2018). Clinical information, including newborn sex and birth weight, gestational age at delivery, maternal age, and ethnicity were collected. Placental and maternal samples were processed and DNA was extracted as previously described (Robinson et al. 2010).

**Fig. 1:**
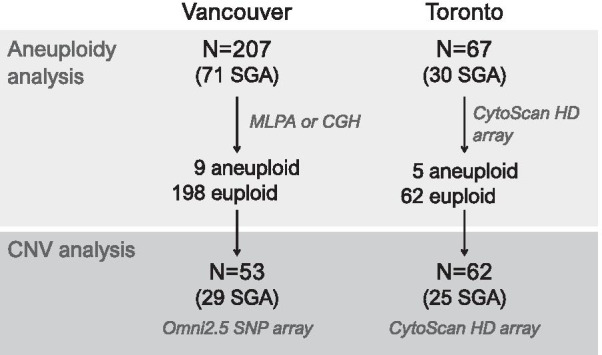
Schematic of the study design, including methods and sample sizes used in both cohorts in this study. Only placentas that were euploid following aneuploidy analysis were used for CNV analysis. Genetic assessment methods are italicized. *CGH* comparative genomic hybridization, *MLPA* multiplexed ligation-dependent probe amplification

This cohort (N = 207) included 136 controls from uncomplicated pregnancies (no SGA, hypertension/PE, or known abnormal maternal serum screen results) and 71 cases of SGA (Table 1). Exclusion criteria were a prenatally-diagnosed chromosome abnormality or congenital anomaly in the fetus. SGA was defined as birth weight < 10th percentile, adjusted for sex and gestational age at birth based on Canadian growth charts (Kramer et al. 2001). The majority, 55/71 (77%) of SGA cases met criteria for FGR, defined as birth weight < 3rd percentile, or < 10th percentile with additional findings suggestive of placental insufficiency, including (i) persistent uterine artery notching at 22–25 weeks, (ii) absent or reversed end diastolic velocity on umbilical artery Doppler, and/or (iii) oligohydramnios (amniotic fluid index < 50 mm). One FGR case had a birth weight > 10th percentile but was diagnosed as FGR from prenatal measurements and severe oligohydramnios. Preeclampsia (PE) was defined according to Canadian criteria (Magee et al. 2014) as previously described (Del Gobbo et al. 2018). A subset of SGA cases were associated with maternal PE (Table 1); the SGA cases associated with maternal PE delivered significantly earlier than those without (mean 33.2 weeks vs. 36.9 weeks, respectively; *p* < 0.05, Mann–Whitney U-test), however birth weight did not differ (*p* > 0.05, Student’s t-test). Following aneuploidy assessment, euploid placentas from a subset of 24 control and 29 SGA cases, 90% of which fulfilled criteria for FGR, were selected for further CNV profiling (Table 1). These were randomly selected after excluding cases or controls associated with a twin pregnancy (N = 23), or known maternal smoking during pregnancy (N = 3/108 respondents). Figure 1 summarizes the study design and number of cases per cohort used at each analysis step.

**Table 1:** Study cohort clinical characteristics

Group	Gestational age at birth (w), mean (range)	Maternal age at birth (y), mean (range)	Sex, N male (%)	Birthweight (S.D.), mean (range)	Twins, N (%)	PE, N (%)
Vancouver cohort—total samples						
Control (N = 136)	39.2 (30.1–41.9)	34.3 (23.8–45.8)	68 (50)	0.1 (− 1.2 to 2.7)	11 (8)	0 (0)
SGA (N = 71)	35.3 (23.6–41.7)*	35.2 (23.1–41.0)	34 (48)	− 1.9 (− 3.6 to − 1.2)*	12 (17)	31 (44)
Subset of samples for CNV profiling						
Control (N = 24)	39.3 (38.0–41.4)	34.8 (30.2–40.5)	13 (54)	0.01 (− 1.1 to 2.2)	0 (0)	0 (0)
SGA (N = 29)	34.9 (24.0–40.6)*	34.4 (23.9–42.9)	18 (62)	− 1.9 (− 3.0 to − 0.6)*	0 (0)	11 (38)
Toronto cohort—total samples						
Control (N = 37)	37.1 (27.3–41.0)	32.9 (21–43)	19 (51)	0.28 (− 1.1–1.5)	0 (0)	0 (0)
SGA (N = 30)	34.0 (27.1–38.6)*	35.1 (25–44)	9 (30)	− 2.2 (− 3.5–1.2)*	5 (17)*	0 (0)

*SGA* small-for-gestational age, *PE* preeclampsia

^*^p < 0.05, *p*-values calculated in comparison to respective control groups by Student’s t-test for maternal age and birth weight, Mann–Whitney U-test for gestational age, and Fisher’s exact test for all categorical variables

#### Toronto cohort

The Toronto cohort was ascertained and processed as part of a distinct study, and findings from the two cohorts were then subsequently compared. Placental samples were obtained as previously described (Ferreira et al. 2011). Clinical information including newborn sex, birth weight, and gestational age were collected for all cases. The original cohort included 99 samples, however following microarray quality filtering, 67 remained, including placentas from 37 control and 30 SGA pregnancies (Table 1, Fig. 1). Definitions for control and SGA followed the same criteria as the Vancouver cohort, described above. Exclusion criteria were a prenatally-diagnosed chromosome abnormality or congenital anomaly in the fetus, CMV or toxoplasmosis infection, or clinical amnionitis. Additionally, cases or controls were excluded if mothers were diagnosed with: (i) preconceptional severe hypertension; (ii) clinically significant thrombophilia; (iii) advanced renal, heart or liver failure; (iv) type I diabetes mellitus or gestational diabetes requiring treatment with insulin; or (v) anemia and autoimmune disorders requiring therapy during pregnancy. Maternal PE was not present in any of the cases in this cohort (Table 1).

### Aneuploidy screening and CPM follow-up

Aneuploidy was detected using several methods in this study. In the Vancouver cohort, samples were assessed by comparative genomic hybridization (CGH), which can detect aneuploidies greater than 15 Mb, or by multiplexed ligation-dependent probe amplification (MLPA) of subtelomeric probes (SALSA MLPA Subtelomeres Mix, MRC-Holland, NL), designed to detect aneuploidies that extend to the ends of the chromosome (Fig. 1). A subset of these samples (N = 85 control and N = 43 SGA), all screened by CGH, have been previously published (Robinson et al. 2010); the current study is a retrospective re-assessment of aneuploidy in those cases, with additional samples collected. For more recent cases, MLPA was used to screen for aneuploidy because it is a reliable and cost-effective method to identify whole chromosome aneuploidies (monosomy and trisomy), as well as terminal duplications and deletions. In the Toronto cohort, aneuploidy was detected using CNV profiling by microarray (see below). All cases with an aneuploidy detected by any method was further assessed by microsatellite polymorphism genotyping of probes on the involved chromosome (Additional file 1: Methods). Aneuploidies identified by MLPA were also confirmed using CNV profiling by microarray to determine the extent of the alteration, particularly in cases where results suggested abnormalities restricted to one chromosome arm (see below, Additional file 1: Methods).

### Microarray processing and CNV detection

Placental DNA was assessed on the Infinium Omni2.5-8 BeadChip array (Illumina, USA) for the Vancouver cohort, and on the Affymetrix CytoScan HD array (ThermoFisher Scientific, USA) for the Toronto cohort (Fig. 1) at The Centre for Applied Genomics, Toronto, Canada (Pinto et al. 2011; Uddin et al. 2015). In the Vancouver cohort, an additional DNA sample from a different location in each placenta was also run on the array to assess the possibility of detecting mosaicism of CNVs by high-density microarray (Additional file 1: Methods). Following sample quality checks unique to each array type, all 54 Vancouver cases and 67/99 Toronto cases were available for analysis (Fig. 1). CNVs were detected using in-house pipelines (Pinto et al. 2011; Uddin et al. 2015) applying 3–4 CNV-calling algorithms specific to each array platform (Additional file 1: Methods). Following CNV quality checks, high-confidence CNVs called by at least two algorithms with a minimum 50% reciprocal overlap, ≥ 5 probes, and ≥ 10 kb were kept for analysis. CNV boundaries were compared to the Database of Genomic Variants and in-house databases of CNVs in controls, and rare CNVs were defined as those present in < 0.1% of controls and at least 50% unique. Given discordance in CNV calls between technical replicates of placental DNA (Additional file 1: Methods, Additional file 3: Figure S1), mosaicism of CNVs was not investigated and the DNA sample with the higher microarray quality scores from each placenta was selected for CNV analysis for the Vancouver cohort. Ancestry was assessed using SNP genotypes by MDS clustering of identity-by-state distances in PLINK (Purcell et al. 2007) (Additional file 1: Methods). The ancestry composition of both cohorts was comparable (Additional file 2: Table S1, Additional file 3: Figure S2).

### Candidate CNVs

CNVs with potential clinical relevance to SGA were prioritized based on: whether they were rare, ≥ 200 kb, overlap pathogenic or likely pathogenic CNVs in the DECIPHER or ClinVar databases, overlap genes with important roles in placental function or those that are reported to be differentially expressed or with variants associated with growth restriction. CNVs were categorized following American College of Medical Genetics guidelines (Kearney et al. 2011). Candidate CNVs were confirmed and assessed for CPM using quantitative PCR (Additional file 1: Methods).

### Placental-enhanced and imprinted genes

A list of 356 genes with elevated expression in the placenta was downloaded from the Human Protein Atlas (https://www.proteinatlas.org/humanproteome/tissue/placenta), including 78 with placental-specific elevated expression. A database of imprinted regions was curated from the OTAGO Imprinted Genes (http://igc.otago.ac.nz/home.html) and GeneImprint (http://www.geneimprint.com/site/genes-by-species.Homo+sapiens) databases, and reported placental imprinted differentially methylated regions (DMRs) (Court et al. 2014; Hanna et al. 2016) (Additional file 1: Table S2). Outer genomic boundaries were used to generate a consensus region for those genes associated with a placental imprinted DMR.

### Functional pathway enrichment

Enrichment of 2191 GO and KEGG (Kanehisa and Goto 2000) pathways in genes with coding sequences impacted by rare CNVs in SGA was assessed using a generalized linear model with universal gene count correction in the *cnvGSA* R package. Sex and cohort (array) were included as covariates, and thresholds of 100–1500 genes were used to limit pathways assessed. A false-discovery rate (FDR) of < 0.1 was used to define significantly enriched (coefficient > 0) or deficient (coefficient < 0) pathways in SGA CNVs.

### Statistical analyses

Continuous variables were compared using the Student’s t-test or Mann–Whitney U test depending on whether the data was normally-distributed by the Shapiro–Wilk normality test. Categorical variables were compared by Fisher’s exact test. Bonferroni correction for multiple testing was used where applicable. Statistical power for comparing CNV load was assessed using the *pwr* package in R. Based on a previous report of a large effect size (*d* > 0.95) in the difference in CNV load in control *vs.* SGA placentas (Kasak et al. 2015), we assumed a slightly lower but still large effect size (*d*) of 0.8. Based on the minimum sample size in each group per cohort (N = 24) and using an α = 0.05, our study had > 80% power to detect significant differences in each cohort individually. Analyses were performed in R version 3.5.1 (R Core and Team 2016), and plots were generated using the *ggplot2*, *ggbio,* and *ggpubr* packages.

## Results

### Poor fetal growth is associated with placental aneuploidy

Aneuploidy screening was performed in 207 placentas from the Vancouver cohort and 67 placentas passing microarray quality checks from the Toronto cohort. Amongst 173 control placentas, no cases of CPM or autosomal aneuploidy were detected. Two (1.1%) controls had constitutional abnormalities involving the sex chromosomes (Table 2), one of which only impacted Yqter. In contrast, amongst 101 SGA cases, 12 (11.9%) had a whole or partial autosomal trisomy present in the placenta (Table 2) (*p* = 0.00017; OR = 11.4, 95% CI 2.5–107.4; Fisher’s exact test). Placental autosomal aneuploidies were found both in cases of isolated SGA (N = 9/70; 12.8%) and cases of SGA with maternal PE (N = 3/31; 9%).

**Table 2:** Summary of findings from detection of placental aneuploidy

Study group (N)	Balanced (M:F)	Unbalanced (M:F)	CGH/MLPA result	Inferred karyotype	CPM
Control (173)	171 (86:85)	2 (1:1)	Gain of X	47, XXX^a^	No
			del(X/Yq)	46,XY,del(Yqter)	Unk
SGA (101)	89 (39:50)	12 (4:8)	Gain of 7	47,XX,+7/46,XX^a^	Yes
			Gain of 7	47,XY,+7/46,XY^a^	Yes
			Gain of 2	47,XX,+2/46,XX^a^	Yes
			Gain of 13	47,XX,+13/46,XX^a^	Yes
			dup(7q),del(Xp)	46,XX,der(X) t(X;7)(p22.2;q21.2)/46,XX	Yes
			del(4q),dup(4p)	46,XY, der(4)del(4)(q34.2), dup(4)(p16.3p15.31)/46,XY	Yes
			Gain of 10, Gain of X	48,XXX,+10/47,XXX	Yes^b^
			N/A	47,XY,+2/46,XY	Unk
			N/A	46,XX,+i(15q)/46,XX	Unk
			N/A	47,XX, + 16/46,XX	Yes
			N/A	47,XX,+16/46,XX	Yes
			N/A	47,XY,+16/46,XY	Yes

*CGH* comparative genomic hybridization, *MLPA* multiplexed ligation-dependent probe amplification, *CPM* confined placental mosaicism, *Unk.* unknown/unable to confirm, *N/A* not available (cases were only screened by microarray)

^a^Cases published in Robinson et al. (2010)

^b^Constitutional trisomy X, CPM of trisomy 10

Of the cases with successful follow-up (10/12), all abnormalities in SGA placentas were determined to be CPM based on microsatellite genotyping (Table 2). Four of these cases were previously published (Robinson et al. 2010), however 8 are new and confirm the association between CPM and SGA. Of the 9 cases with available maternal DNA, uniparental disomy (UPD) was excluded in the diploid cell population from all but one previously-published case with CPM for trisomy 2 and probable upd(2)mat (Robinson et al. 2010). The incidence of aneuploidy did not differ between cohorts (2/136 vs. 0/37 controls and 7/71 vs. 5/30 SGA in the Vancouver and Toronto cohorts, respectively). Overall, our cohorts had high maternal ages (Table 1), and among the SGA cases, maternal age tended to be higher in pregnancies associated with CPM than those without a placental aneuploidy (Additional file 2: Table S3), though this was not significant (*p* > 0.05, Student’s t-test).

### Load of CNVs does not differ between SGA and control placentas

To explore the role of placental CNVs in in utero growth, 114 euploid placentas from control and SGA newborns were assessed using high-density microarrays (Fig. 1). We found one SGA case (PM324) with mosaicism for 8 large 2–4 Mb duplications in the placenta (Additional file 3: Figure S3). As the combined level of aneuploidy exceeded 27 Mb, it was an outlier that was excluded it from further comparisons, so as to not bias results; we instead considered it as an additional case of placental segmental aneuploidy. Due to significant differences in load of CNVs between the different array platforms (Additional file 2: Table S4), we performed case–control comparisons within each cohort independently. We found no difference in total number and cumulative extent (bp) of CNVs per placenta, except for a greater cumulative bp of rare CNVs in SGA placentas in the Vancouver cohort (*p* = 0.03, Mann–Whitney U test) (Table 3). When comparing these measures by gains and losses separately, there were also no significant differences (Table 3).

**Table 3:** Summary of load of CNVs in control and SGA placentas

	Vancouver cohort		Toronto cohort	
	Control (N = 24)	SGA^a^ (N = 28)	Control (N = 37)	SGA (N = 25)
N CNVs	17 (11–25)	16 (9–27)	35 (20–57)	32 (22–52)
Gains	7 (1–11)	7 (1–14)	17 (9–38)	15 (8–36)
Losses	10 (4–20)	9 (3–17)	18 (9–33)	17 (10–29)
N rare CNVs	4 (1–10)	4 (2–10)	7 (1–29)	6 (1–19)
Gains	1 (0–6)	2 (0–6)	4 (0–22)	3 (0–16)
Losses	3 (0–7)	3 (0–9)	3 (0–12)	3 (0–6)
Cumul. size (Mb)	1.22 (0.44–3.43)	1.59 (0.57–5.12)	3.20 (1.25–7.86)	3.17 (1.63–5.80)
Gains	0.57 (0.03–1.32)	0.86 (0.03–2.85)	2.36 (1.06–6.97)	2.05 (0.97–5.11)
Losses	0.65 (0.06–2.92)	0.72 (0.11–3.45)	0.84 (0.18–2.33)	1.12 (0.36–4.19)
Cumul. size rare (kb)	219 (10–902)	327 (74–864)*	893 (38–3652)	825 (19–3329)
Gains	100 (0–781)	197 (0–819)	678 (0–3460)	516 (0–2937)
Losses	119 (0–485)	130 (0–517)	1570 (0–5776)	1373 (0–4516)

*Cumul.* cumulative

*p < 0.05, Mann–Whitney U-test. All values reported as mean (range)

^a^Excludes outlier PM324

As larger CNVs are more likely to be impactful, we compared CNV size across all placentas in each group. In the Vancouver cohort, CNVs were larger in SGA placentas (*p* = 0.002, Mann–Whitney U test; Additional file 3: Figure S4). When considering CNV gains and losses separately, only the losses were significantly larger (*p* = 0.010, Mann–Whitney U test; Additional file 3: Figure S4). When separated by sex, the larger CNV sizes in SGA were significant only amongst females (Additional file 3: Figure S5). There were no significant differences between groups in the Toronto cohort.

**Fig. 2:**
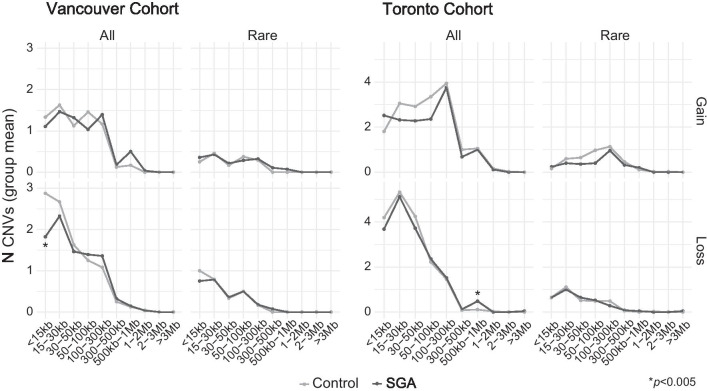
Sizes of placental CNVs from control and SGA pregnancies. Plots depict the mean number of CNVs per study group at different size bins in the Vancouver and Toronto cohorts, in all CNVs or exclusively rare CNVs, and separated by gains and losses. Overall, there is no consistent difference in the sizes of CNVs between SGA and control placentas. *p*-values calculated by Mann–Whitney U-test

To further assess whether SGA placentas had a greater CNV load, we compared the number of gains or losses per placenta at size bins ranging from < 15 kb to > 3 Mb in all CNVs or only in rare CNVs between groups. There were no consistent differences between SGA and control placentas. SGA placentas in the Vancouver cohort had fewer small losses (< 15 kb, *p* = 0.002; Mann–Whitney U-test), and those in the Toronto cohort had more large losses (500 kb–1 Mb, *p* = 0.001; Mann–Whitney U-test). Both of these findings withstood multiple test corrections at a Bonferroni-corrected *p*-value threshold of *p* = 0.005, but were not observed in rare CNVs (Fig. 2).

### Candidate CNVs identified in SGA placentas

We next focused on rare CNVs ≥ 200 kb as these are most likely to contribute to the SGA phenotype. There were 34 large rare CNVs present in SGA placentas and 53 in controls. CNVs with potential roles in placental function and/or fetal growth were identified 5.7% (3/53) of SGA placentas but not in controls (0/61). The SGA cases carrying a candidate CNV were all isolated SGA without maternal PE. The three candidate CNVs were categorized as variants of uncertain significance (VUS)-likely pathogenic and impact the functionally relevant genes *IHNBB, HSD11B2*, *CTCF*, and *CSMD3* (Table 4). These were confirmed by qPCR to be present in both placenta and cord blood, thus were not confined to the placenta.

**Table 4:** Candidate CNVs with clinical relevance to SGA identified in study placentas

Case ID	Sex	Study group	Genomic coordinates (hg19)	Size (kb)	CNV type	Genes	Category	CPM
7665	Female	SGA	2:121,092,278–121,914,455	822	Gain	*INHBB*, *GLI2*	VUS-likely pathogenic	No
6234	Female	SGA	16:67,150,183–67,615,830	466	Loss	*HSD11B2*, *CTCF*, 21 others	VUS-likely pathogenic	No
10506	Female	SGA	8:112,947,262–116,124,691	3177	Loss	*CSMD3*	VUS-likely pathogenic	No

*CPM* confined placental mosaicism, *VUS* variant of uncertain significance

### No difference in total, placental-enhanced, or imprinted genes impacted by placental CNVs

**Fig. 3:**
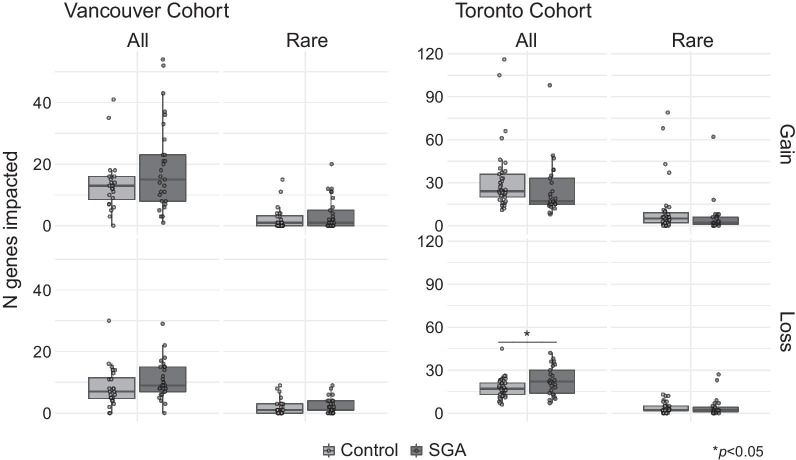
Total number of genes impacted by placental CNVs from control and SGA pregnancies. The cumulative total of unique RefSeq genes impacted by CNVs for each case in the Toronto and Vancouver cohorts are shown, separated by all CNVs or exclusively rare CNVs, and by gains and losses. Toronto cohort SGA placentas had slightly more genes affected by losses than controls. A similar trend was found in Vancouver cohort, but the difference was not statistically significant. *p*-values calculated by Mann–Whitney U-test

To investigate potential impact of CNVs, we compared the number of genes involved in CNVs per case. We found no differences in the Vancouver cohort, however there was a trend for a greater number of genes affected by losses in SGA placentas in the Toronto cohort (*p* = 0.049, Mann–Whitney U-test; Fig. 3). There were no significant differences when focusing on rare CNVs.

We did not find an enrichment of genes with enhanced placental expression in SGA CNVs, however there were more losses of placental-enhanced genes in controls in the Toronto cohort (*p* = 0.02, Fisher’s exact test; Additional file 2: Table S5) that was not reproduced in the Vancouver cohort. Gains impacting *ERVV-1* and *ERVV-2*, and CNVs impacting several *PSG* family genes, a region known to be copy number variable in the human population (Chang et al. 2013), were common in both cases and controls.

We did not find any significant enrichment of imprinted regions in placental CNVs from SGA cases (Additional file 2: Table S6). Several common CNVs impacting imprinted regions were recurrent, including placental imprinted DMRs for *SPRN* and *CYP2E1* (Additional file 1: Table S7). CNVs deemed as rare were also recurrent, including gains impacting *KCNK9* and the DMR near *PRMT2* (Additional file 1: Table S7). One rare CNV was present uniquely in a SGA case, arr[hg19] 22q11.21(19,931,668–19,980,300) × 1, overlapping the placental-specific imprinted DMR and coding sequence of *ARVCF*. One other CNV resulted in a deletion of the growth-related gene *INS* in a control: arr[hg19] 11q15.5(2,170,670–2,199,458) × 1 (Additional file 2: Table S7).

### No significantly enriched gene pathways in SGA CNVs

Out of 1872 GO and KEGG pathways with genes involved in rare CNVs, we did not find any significantly enriched pathways in SGA CNVs (FDR > 0.4). 8 pathways were enriched at a nominal *p* < 0.05, the top being “negative regulation of cell cycle” (*p* = 0.031), and 7 were deficient (Additional file 2: Table S8). Investigating gains and losses separately, no enriched pathways were identified (FDR > 0.4). 10 pathways were enriched in SGA gains at a nominal *p* < 0.05, the top being “regulation of cellular response to stress” (*p* = 0.009), and three pathways were deficient in SGA gains (Additional file 2: Table S8).

## Discussion

In this study, we investigated the contribution of genomic imbalances in the placenta to poor fetal growth. In our otherwise low-risk population, we found that CPM involving trisomy or large segmental aneuploidy was present in 11.9% of SGA cases, or 12.7% when including the case with duplications totaling > 27 Mb. Placental aneuploidy was present at similar rates in SGA whether or not maternal PE was also present (isolated SGA: 12.8%, SGA with PE: 9.7%), although a greater sample size is needed to accurately compare these incidences. The significant association of trisomy CPM to SGA/FGR confirms previous reports (Artan et al. 1995; Krishnamoorthy et al. 1995; Wilkins-Haug et al. 1995; Stipoljev et al. 2001; Robinson et al. 2010), however we have additionally identified cases of CPM of large segmental aneuploidies contributing to SGA, including a dup(7)(q21.2q36.3), del(X)(p22.2) likely deriving from a X;7 translocation event, and a case with dup(4)(p16.3p15.31), del(4)(q34.2). Although CPM can occur in healthy pregnancies (Wilkins-Haug et al. 1995; Wapner et al. 1992; Fryburg et al. 1993; Toutain et al. 2010), only non-mosaic aneuploidies affecting the sex chromosomes were identified in our controls.

The incidence of placental aneuploidy associated with SGA in this study is comparable to past reports (Krishnamoorthy et al. 1995; Stipoljev et al. 2001; Robinson et al. 2010), however it is expected to be population-dependent. The frequency of trisomy, and thus CPM, increases with advanced maternal age, which is also a risk factor for SGA. Indeed, we found that maternal age tended to be higher in SGA pregnancies with CPM (mean: 36.7 y) than those that were chromosomally-balanced (mean: 35.0 y). Conversely, CPM should contribute to fewer cases of SGA in populations with high rates of other risk factors for SGA, such as maternal smoking or poor nutrition (Blatt et al. 2015; Black et al. 2013). A higher CPM incidence is also expected using a stricter definition of FGR rather than SGA, e.g. fetal weight < 3rd percentile or by using biomarkers like placental growth factor (PlGF) in maternal serum that are predictive of placental-mediated FGR (Benton et al. 2016). Although we could not measure maternal PlGF levels, our SGA group was likely enriched for cases of pathological growth restriction as a large proportion of cases were < 3rd percentile (68% Toronto cohort, 48% Vancouver cohort) and the majority of cases in the Vancouver cohort met criteria for FGR.

Overall, we could not confirm previous reports finding decreased (Kasak et al. 2015) or increased (Biron-Shental et al. 2016) load of CNVs in SGA placentas compared to controls. Small sample size may explain these discrepancies, as both past studies had < 10 cases per group. With greater sample size and low incidence of other risk factors in our population, we were well poised to detect genetic contributors to SGA. Although we identified trends that suggest that some SGA placentas have an increased load of large CNVs, our findings did not support that placental CNVs commonly contribute to SGA. We also did not find significant differences in number of total or placental-expressed genes or imprinted regions in CNVs, which also suggests that either these are not major drivers of poor fetal growth in our cohort or their effects are subtler than we had power to detect.

Nonetheless, a candidate VUS-likely pathogenic germline CNV was identified in 5.7% of SGA placentas in this study, all of which were SGA in the absence of maternal PE. This incidence is similar to past studies of prenatal samples, which identified pathogenic CNVs in 3–7% of cases of isolated FGR with normal karyotypes (Zhu et al. 2016; Borrell et al. 2017; Shaffer et al. 2012). Case 7665 has a duplication of *INHBB*, which encodes a subunit for the activin and inhibin proteins that play important roles in trophoblast growth and invasion (Bearfield et al. 2005; Li et al. 2014), and altered mRNA or protein levels of these compounds are associated with miscarriage, severe PE, and FGR (Wijayarathna and Kretser 2016). Case 6234 has a deletion encompassing *HSD11B2* and part of *CTCF*. *HSD11B2* is highly expressed in placental trophoblast cells, and encodes 11*β*-HSD2, which regulates fetal exposure to maternal glucocorticoids (Krozowski et al. 1995). Reduced placental *HSD11B2* gene expression or protein levels has been associated with FGR (McTernan et al. 2001; Dy et al. 2008; Zhao et al. 2014; Lazo-de-la-Vega-Monroy et al. 2017), and patients with rare mutations in *HSD11B2* have significantly lower birth weight (Dave-Sharma et al. 1998). *CTCF* is a highly-conserved transcription factor, and rare loss-of-function variants or deletions of the gene are associated with low birth weight, postnatal growth retardation, microcephaly and intellectual disability (Gregor et al. 2013). Case 10506 had a 3 Mb deletion encompassing *CSMD3*, which is reported to be intolerant to loss-of-function variants [upper bound o/e = 0.3 in gnomAD (Karczewski et al. 2020)], and *Csmd3* knockout mice display lower body length and body fat (Bult et al. 2019).

### Strengths and limitations

This is the first study to our knowledge to characterize both aneuploidy and copy number variants in the placenta in association with poor fetal growth. It also contributes the largest sample evaluated for the association between placental CNVs and SGA to date. This CNV assessment was comprehensive, as we incorporated rigorous data processing following well-established pipelines, and several thorough lines of investigation to establish the copy number profile of the placenta in association with SGA, as well as potential clinical relevance of CNVs to poor fetal growth.

Due to the retrospective nature of this study, differences exist in clinical characteristics and methodologies between the cohorts and are a limitation of the study. Certain exclusion criteria used in the Toronto cohort were not available in the Vancouver cohort (e.g. infection during pregnancy), therefore we could not exclude such cases. Additionally, some cases of SGA in the Vancouver cohort were associated with maternal PE whereas all Toronto cohort cases were of isolated SGA. Aneuploidy screening methods used were also not equivalent, as MLPA cannot detect large interstitial duplications or deletions. Despite this, the Vancouver and Toronto cohorts had similar clinical characteristics (Table 1, Additional file 2: Table S1) and the methods to screen for aneuploidy all accurately identify whole chromosome or chromosome arm abnormalities, therefore we combined the cohorts to improve our power to establish the contribution of placental aneuploidy to SGA. A limited number of placental biopsies were used to screen for aneuploidy in both cohorts, therefore it is likely that aneuploidies present at low levels or in a limited distribution in the placenta were missed.

Unlike the aneuploidy assessment, we were unable to combine the two cohorts to study CNV load associated with SGA due to the significant differences between the high-density microarrays used for CNV detection. However even when assessed separately, each cohort had adequate power to identify differences at the large effect sizes described in previous reports (Kasak et al. 2015; Biron-Shental et al. 2016), and testing the two cohorts independently gave us the opportunity to assess the reproducibility of our findings.

### Research and clinical implications

An appreciation for the association between placental aneuploidy and SGA/FGR is relevant for both research and clinical applications. For studies investigating the etiology of idiopathic SGA/FGR, excluding cases explained by CPM may increase the power of association studies. When identified prenatally, CPM may signify that the pregnancy is at increased risk for complications depending on the extent of the abnormality and the chromosome(s) involved. For example, CPM of trisomy 8 has low risk of complications (Cassina et al. 2018), while that of trisomy 16 is associated with a high risk for FGR and PE (Robinson et al. 1997; Yong et al. 2006, 2003; Benn 1998). There is also an increased risk of UPD in the diploid cell population, which can be associated with imprinting disorders; for example, upd(7)mat and upd(20)mat are associated with FGR and several long-term health complications (Kotzot et al. 1995; Mulchandani et al. 2016). Reassuringly, follow-up studies of cases of CPM without UPD suggest that most growth-restricted infants tend to have catch-up growth, normal neurodevelopment, and no global developmental delay (Fryburg et al. 1993; Amor et al. 2006; Langlois et al. 2006; Sparks et al. 2017). Identifying cases that were growth-restricted due to CPM can inform further long-term outcome studies, particularly in relation to specific trisomies, to improve our understanding of the developmental trajectories and risks for complications in affected infants, and address the clinical utility of screening for CPM and UPD in cases of FGR.

Our findings also provide evidence that CNVs impacting genes relevant to growth or placental function may contribute to idiopathic SGA. In contrast to findings of aneuploidy CPM, the CNVs identified in our study were germline alterations and may therefore have clinical implications beyond birth. Future studies profiling CNVs associated with SGA or FGR may add to ours and improve the annotation of CNVs found in cases of obstetric complications, for which information is largely absent in population databases. Given the widespread use of non-invasive methods to detect placental DNA in maternal blood and the development of methods to identify CNVs from these samples (Chen et al. 2013; Wapner et al. 2015; Yin et al. 2015), the feasibility of identifying pathogenic CNVs prenatally is increasing. This will have relevant implications for both predicting pregnancies at risk of FGR and its associated complications and for post-natal counselling if CNVs are not confined to the placenta. Additional research on the incidence and impact of CNVs on obstetric outcomes is thus needed to assess the potential clinical utility of this information.

## Conclusions

Overall, we find consistent evidence that trisomy and segmental aneuploidy confined to the placenta are associated with a significant proportion of cases of poor fetal growth, and that rare germline CNVs overlapping genes of functional interest may also underlie a subset of idiopathic SGA cases. Together, these genomic imbalances may explain approximately 18% of SGA cases in our study population, and additional studies to evaluate the clinical utility of screening for these abnormalities are warranted. Increased placental CNV load may not commonly impact fetal growth, however studies with larger sample sizes may help elucidate whether subgroups of SGA/FGR are linked to placental CNV load.

## Supplementary Information

**Additional file 1.** Additional Methods.

**Additional file 2.** Additional Tables S1–S8.

**Additional file 3.** Additional Figures S1–S5.

## Data Availability

The data that support the findings of this study are available from the corresponding author upon reasonable request.

## Abbreviations

CGH: Comparative genomic hybridization;
CNV: Copy number variant
CPM: Confined placental mosaicism
DMR: Differentially methylated region
FDR: False-discovery rate
FGR: Fetal growth restriction
GO: Gene Ontology
KEGG: Kyoto Encyclopedia of Genes and Genomes
MDS: Multidimensional scaling
MLPA: Multiplexed ligation-dependent probe amplification
PE: Preeclampsia
PlGF: Placental growth factor
SGA: Small-for-gestational age
SNP: Single nucleotide polymorphism
UPD: Uniparental disomy
VUS: Variant of uncertain significance
