# Supplementary material for: Genomic imbalances in the placenta are associated with poor fetal growth
Source: Mol Med. 2021 Jan 7;27:3. doi: 10.1186/s10020-020-00253-4 (PMC7792164; doi:10.1186/s10020-020-00253-4)
Supplement: Supplementary file 1 — Additional file 1. Additional Methods. [file 10020_2020_253_MOESM1_ESM.pdf]

Placental genomic imbalances contribute to poor fetal growth

Giulia F. Del Gobbo, Yue Yin, Sanaa Choufani, Emma A Butcher, John Wei, Evica Rajcan-Separovic, Hayley Bos, Peter von Dadelszen, Rosanna Weksberg, Wendy P. Robinson, Ryan K.C. Yuen

Supplementary Methods

Aneuploidy screening

DNA from at least two distinct locations from each placenta from the Vancouver cohort were screened for aneuploidy using comparative genomic hybridization (CGH) as previously described[1], or multiplexed ligation-dependent probe amplification (MLPA) of subtelomeric probes on each chromosome (SALSA MLPA Subtelomeres Mix, MRC-Holland, NL). Cases with a suspected aneuploidy by MLPA were assessed using the Infinium Omni2.5-8 BeadChip array (Illumina, USA; see below). The extent of the aneuploidy was confirmed by inspecting probe intensities (LRR) and allele frequencies (BAF) of involved chromosomes and employing the *cnvPartition* algorithm in GenomeStudio 2.0 (Illumina). Aneuploidy screening in the Toronto cohort was performed alongside CNV profiling by high-density microarray (see below).

Confined placental mosaicism follow-up

Mosaicism of aneuploidies was determined by genotyping microsatellite polymorphisms on involved chromosomes and comparing allelic ratios in all available tissues associated with the placenta[1]. An aneuploidy was determined to be confined to the placenta if it was not detectable in the amnion and/or umbilical cord for cases from the Vancouver cohort, or in cord blood for cases from the Toronto cohort. Maternal blood or decidual contamination of placental samples is not expected based on our sampling techniques, and this was confirmed by comparison to maternal alleles from blood or decidua where available. Confirmation and assessment of mosaicism of CNVs was determined by quantitative PCR in placental and cord blood DNA. All mosaicism follow-up for the Toronto cohort samples was performed at The Centre for Applied Genomics, Hospital for Sick Children, Toronto, Canada.

Microarray processing and sample filtering

Placental DNA from two distinct locations in the same placenta (biological replicates) were run on the Infinium Omni2.5-8 BeadChip array (Illumina, USA) for cases from the Vancouver cohort. 6 sets of technical replicates

were also run to assess technical variability. For the Toronto cohort, one DNA sample from each placenta was run on the Affymetrix CytoScan HD array (ThermoFisher Scientific, USA). For two sets of monozygotic (MZ) twins in the Toronto cohort, DNA from one sample from each twin's share of the same placenta were assessed, serving as biological replicates. All microarrays were processed at The Centre for Applied Genomics following established protocols[2,3]. Poor quality samples were detected and removed if they had i) call rate < 0.97, ii) Log R Ratio (LRR) SD >0.3, or iii) waviness factor > 0.04 (Vancouver cohort); or if they failed Affymetrix default quality filters of "waviness_sd" or "SNPQC" (Toronto cohort). Significant maternal contamination in a villus sample would result in shifted allele frequencies and low quality scores, thus any affected sample would be filtered.

CNV detection and quality checks

CNVs were detected using in-house pipelines[2,3], using CNV-calling algorithms designed for the different array platforms: iPattern[2], PennCNV[4], and QuantiSNP[5] for the Infinium array (Vancouver cohort); and iPattern, Nexus[6], Partek[7], and Chromosome Analysis Suite (ThermoFisher Scientific) for the Affymetrix array (Toronto cohort). Due to limitations of the algorithms, large CNVs are often fragmented, therefore all large CNVs and all chromosomes with >1 Mb of CNVs in a sample were manually inspected. If a CNV was found to be fragmented, the calls were merged and boundaries confirmed by inspecting the probe intensities (LRR) and allele frequencies (BAF) in the region. Samples with excessive CNV calls (> mean + 3 s.d.) were removed. Samples with an aneuploidy detected by CNV profiling in the Toronto cohort were excluded from further CNV analysis. High-confidence CNVs called by at least two algorithms with a minimum 50% reciprocal overlap, ≥5 probes, and ≥10 kb were kept for analysis.

To assess the potential for detection of mosaicism of CNVs in the placenta, concordance in CNV calls between biological and technical replicates was assessed. Concordance between replicate A and B was defined as:

$$Concordance = \frac{N\ overlap\ AB}{N\ overlap\ B + N\ unique\ A + N\ unique\ B}$$

The range of concordance of CNVs between biological replicates was 0.35-0.94 (mean: 0.73) and between technical replicates was 0.64-0.92 (mean: 0.79) (Additional file 3: Figure S1). The majority (67%) of discordant CNV calls between technical replicates were deemed non-stringent in one replicate but stringent in the other. The

rest were not detected by any algorithm in one replicate, but by at least two (stringent CNV) in the other. Given the lack of concordance between technical replicates, only one DNA sample per placenta was selected for further CNV studies. Additionally, one twin per MZ twin pair was also excluded from further studies to ensure a random sample.

Ancestry Assessment and Population Stratification

Ancestry was assessed using SNP genotypes from the microarray experiments in PLINK[8] (Vancouver: v1.09, Toronto: v1.07) using the MDS clustering of identity-by-state distances independently in both cohorts. Probes mapping to the sex chromosomes, those with a call rate <0.95, or with a minor allele frequency <0.05 (Vancouver: 1,085,958; Toronto: 557,487) were removed. LD pruning was performed with a window size=50 kb, step size=5, and r^2=0.25, and population stratification using MDS clustering of identity-by-state distances in PLINK was performed on the resulting in 163,089 and 115,465 tag SNPs for the Vancouver and Toronto cohorts, respectively. Values for the top 10 MDS coordinates were visualized in R and used to describe ancestry as a continuous variable. The top three MDS coordinates captured the majority of variation in ancestry between samples in both cohorts, separated the main ancestry groups into clusters (Additional File 3: Figure S2). Population stratification was tested using the Kolmogorov-Smirnov test between cases and controls for each of the top three coordinates. The Vancouver SGA group had significantly more individuals with East Asian ancestry (Coordinate 1 p=0.031), and the Toronto cohort had significantly more individuals with East Asian (Coordinate 1 p=0.009) and South Asian (Coordinate 2 p=0.030) ancestry in the SGA group (Additional file 3: Figure S2). Overall, the ancestry composition of the two cohorts was comparable (Additional file 2: Table S1).

References

1. Robinson WP, Penaherrera MS, Jiang R, et al. Assessing the role of placental trisomy in preeclampsia and intrauterine growth restriction. *Prenat Diagn*. 2010;30(1):1-8. doi:10.1002/pd.2409

2. Pinto D, Darvishi K, Shi X, et al. Comprehensive assessment of array-based platforms and calling algorithms for detection of copy number variants. *Nat Biotechnol*. 2011;29(6):512-520. doi:10.1038/nbt.1852

3. Uddin M, Thiruvahindrapuram B, Walker S, et al. A high-resolution copy-number variation resource for clinical and population genetics. *Genet Med Off J Am Coll Med Genet*. 2015;17(9):747-752. doi:10.1038/gim.2014.178

4. Wang K, Li M, Hadley D, et al. PennCNV: an integrated hidden Markov model designed for high-resolution copy number variation detection in whole-genome SNP genotyping data. *Genome Res*. 2007;17(11):1665-1674.

5. Colella S, Yau C, Taylor JM, et al. QuantiSNP: an Objective Bayes Hidden-Markov Model to detect and accurately map copy number variation using SNP genotyping data. *Nucleic Acids Res*. 2007;35(6):2013-2025. doi:10.1093/nar/gkm076

6. Darvishi K. Application of Nexus copy number software for CNV detection and analysis. *Curr Protoc Hum Genet*. 2010;Chapter 4(Journal Article):Unit 4.14.1-28. doi:10.1002/0471142905.hg0414s65

7. Downey T. Analysis of a multifactor microarray study using Partek genomics solution. *Methods Enzymol*. 2006;411(Journal Article):256-270.

8. Purcell S, Neale B, Todd-Brown K, et al. PLINK: a tool set for whole-genome association and population-based linkage analyses. *Am J Hum Genet*. 2007;81(3):559-575.
