# Supplementary material for: Genomic imbalances in the placenta are associated with poor fetal growth
Source: Mol Med. 2021 Jan 7;27:3. doi: 10.1186/s10020-020-00253-4 (PMC7792164; doi:10.1186/s10020-020-00253-4)
Supplement: Supplementary file 3 — Additional file 3. Additional Figures S1–S5. [file 10020_2020_253_MOESM3_ESM.pdf]

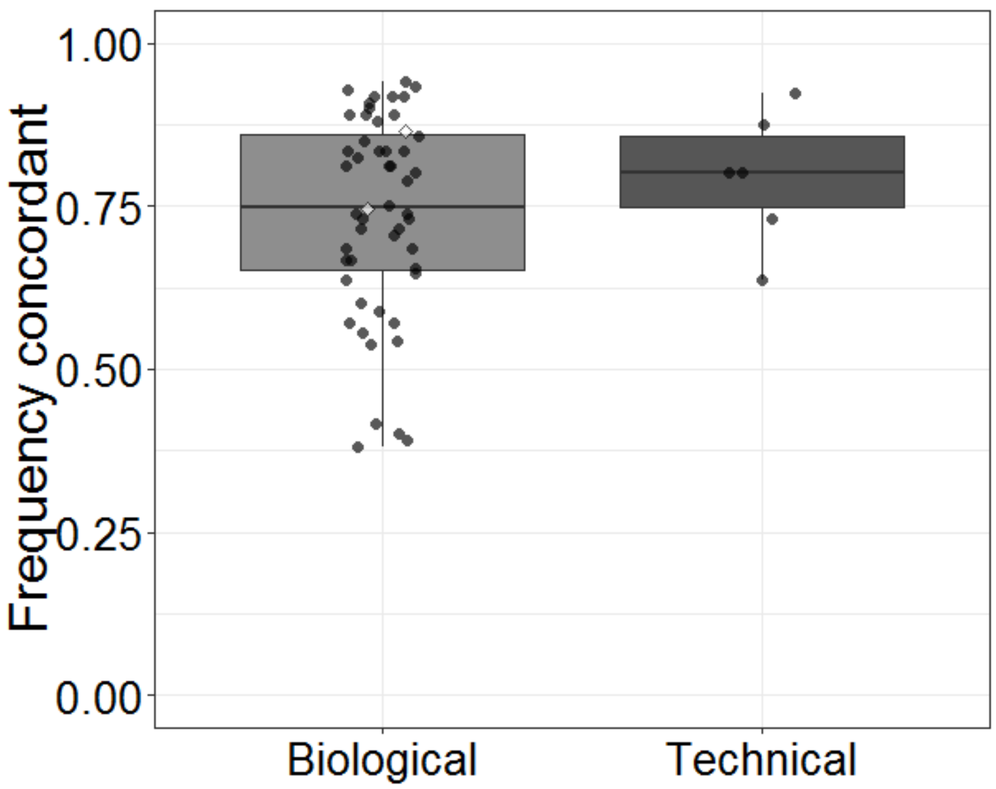


**Figure S1.** Concordance in high-confidence CNV calls between biological or technical replicates of placental DNA. Values for biological or technical replicates from the Vancouver cohort are indicated as black dots, and biological replicates from each monozygotic twins' share of the placenta from the Toronto cohort are indicated by white diamonds.

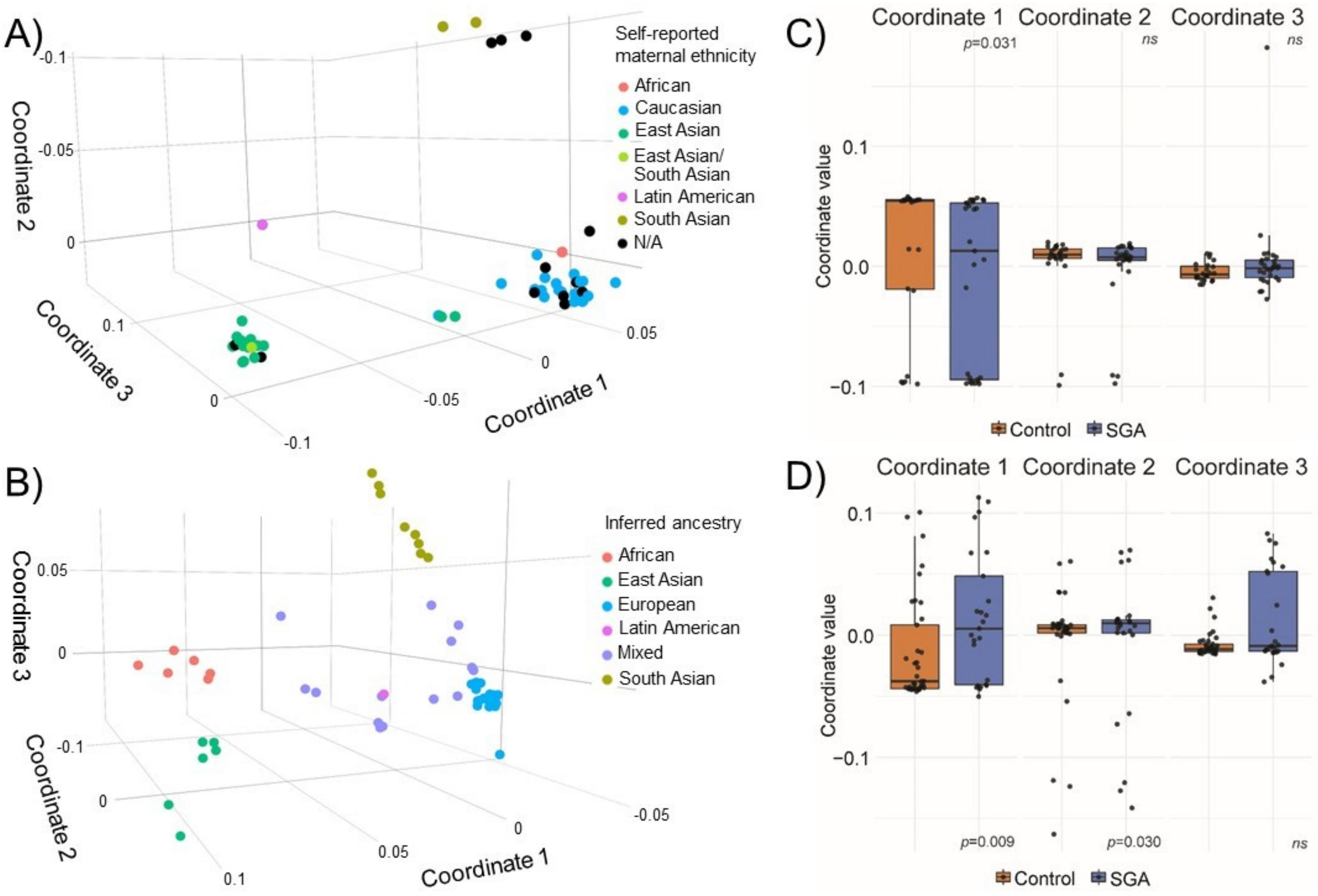


**Figure S2.** Ancestry assessment in placental DNA from two Canadian cohorts. Ancestry in the Vancouver (**A**) and Toronto (**B**) cohorts are represented by the top 3 MDS coordinates, inferred using maternal self-reported ethnicity for available cases. Population stratification between SGA and controls was assessed by comparison of the top three MDS coordinates in the Vancouver (**C**) and the Toronto (**D**) cohorts independently. Vancouver cohort coordinate 1 value is significantly lower in SGA cases, indicating a greater number of individuals with East Asian ancestry. Toronto cohort coordinates 1 and 2 are significantly different in SGA compared to controls, representing the higher number of individuals with East and South Asian ancestry in the SGA group.

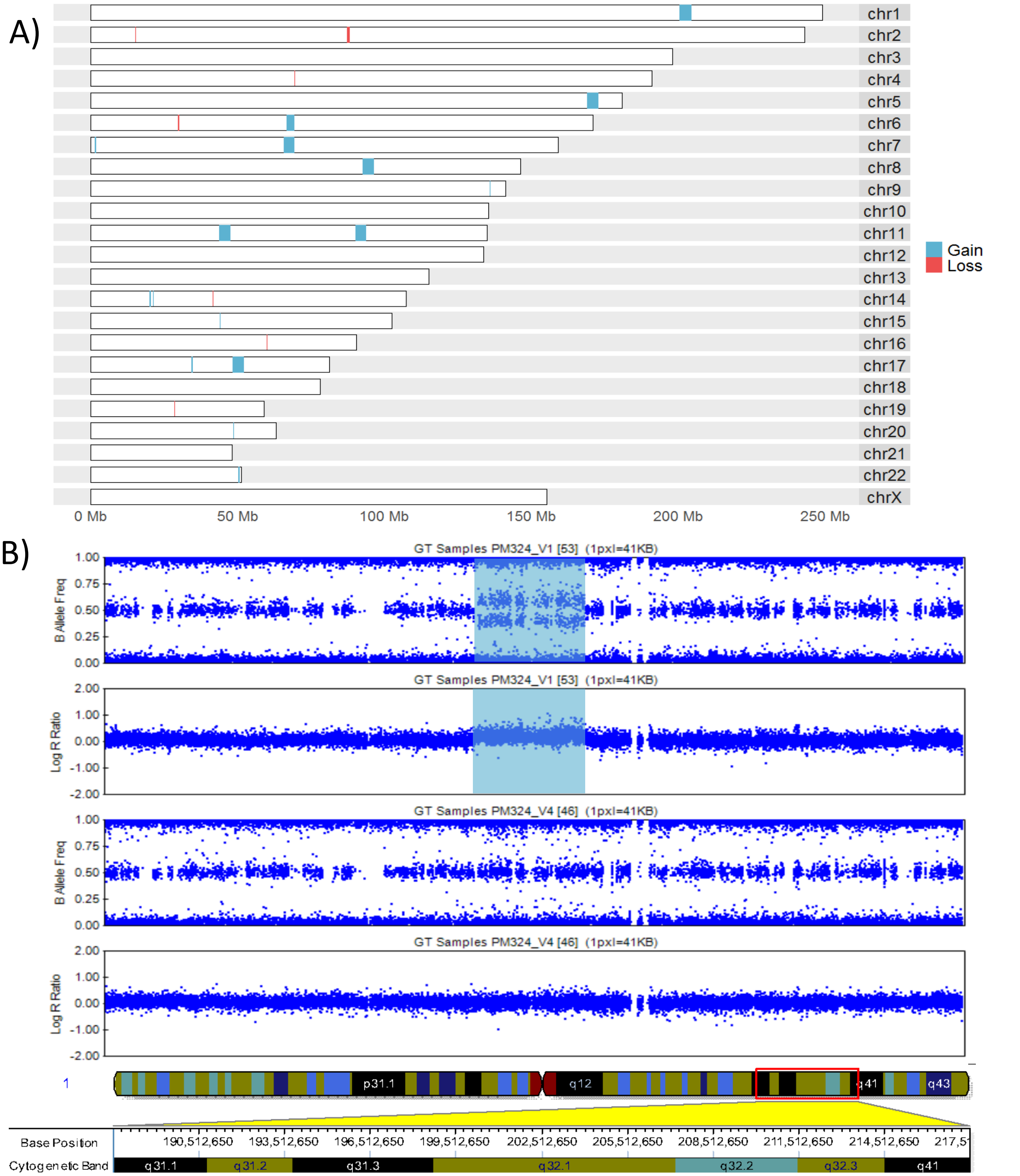


**Figure S3.** A case of placental mosaicism of large CNVs identified by SNP array. **A)** Genomic distribution of CNVs in PM324 placental site V1. Eight large duplications 2-4 Mb in size were identified in seven chromosomes, amounting to 27.4 Mb, in one of two DNA samples from the placenta. **B)** Illumina Genome Viewer image of both sites of placental DNA from PM324 showing the B Allele Frequency (BAF) and Log R Ratio (LRR) for the region containing one of the duplications at chr1:200,478,352-204,413,297 (hg19). Shifts in BAF and LRR are indicative of a mosaic duplication in site V1 (top), which is absent from site V4 (bottom).

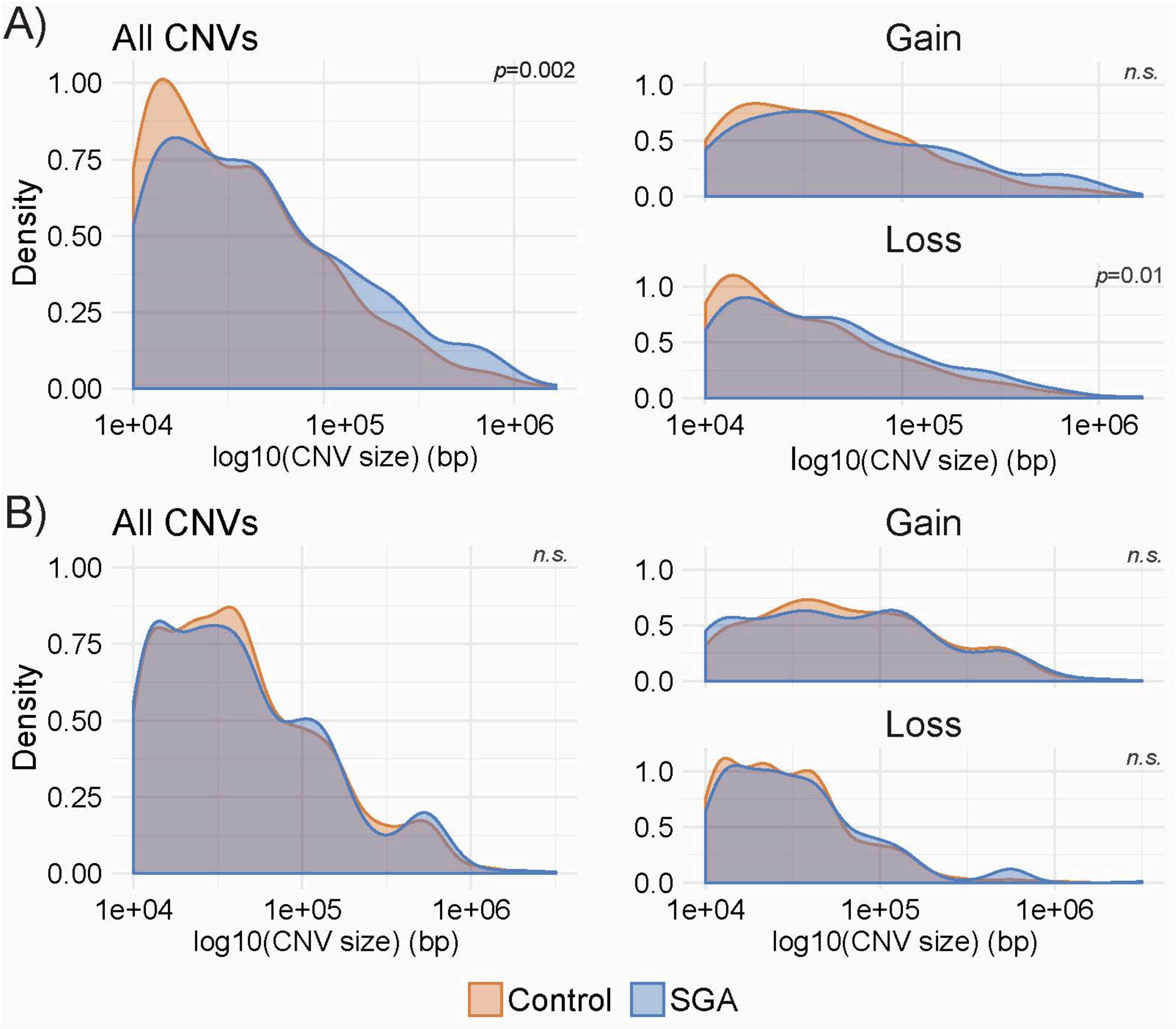


**Figure S4**. Comparison of sizes of all placental CNVs between SGA and controls in the Vancouver **(A)** and Toronto **(B)** cohorts. Vancouver cohort SGA placentas have significantly larger CNVs than controls. Separating by CNV type, this was only significant for losses. There were no significant differences between overall CNV sizes in the Toronto cohort SGA and control placentas. *p*-values calculated by Mann-Whitney U-test.

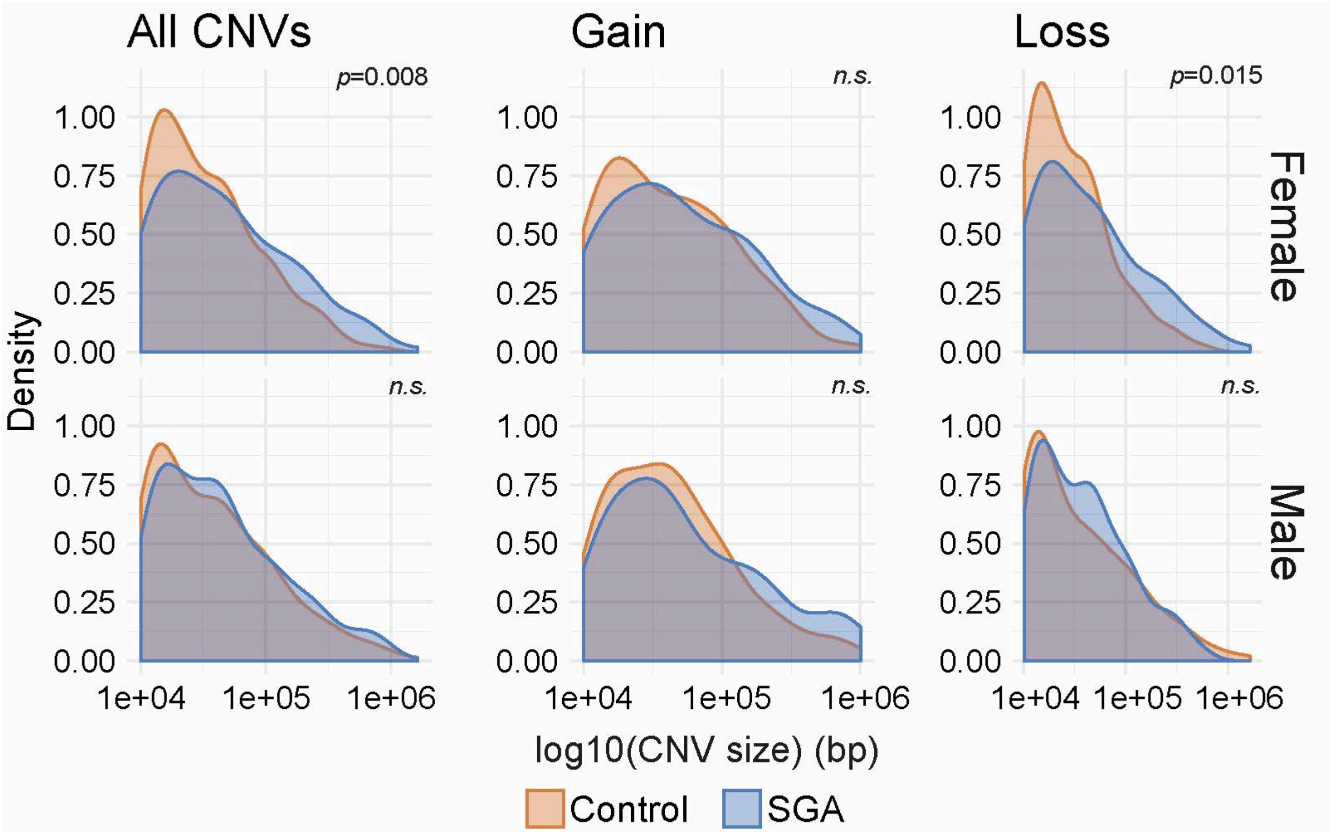


**Figure S5.** Differences in CNV sizes between SGA and controls are present in placentas from females but not males in the Vancouver cohort**.** Density distributions of sizes of CNVs in male and female SGA compared to control placentas. Placentas from SGA females had significantly larger CNVs compared to female controls. In particular placentas from females with SGA had significantly larger losses than female controls. No significant differences were identified between sizes of CNVs in male SGA *vs* control placentas. *p*-values calculated by Mann-Whitney U test.
